# Digital polymerase chain reaction for detecting *c-MYC* copy number gain in tissue and cell-free plasma samples of colorectal cancer patients

**DOI:** 10.1038/s41598-018-38415-4

**Published:** 2019-02-07

**Authors:** Kyu Sang Lee, Soo Kyung Nam, Soo Hyun Seo, Kyoung Un Park, Heung-Kwon Oh, Duck-Woo Kim, Sung-Bum Kang, Woo Ho Kim, Hye Seung Lee

**Affiliations:** 1 Department of Pathology, Seoul National University Bundang Hospital, 173-82 Gumi-ro, Bundang-gu, Seongnam-si, Gyeonggi-do 13620 Republic of Korea; 2 Department of Laboratory Medicine, Seoul National University Bundang Hospital, 173-82 Gumi-ro, Bundang-gu, Seongnam-si, Gyeonggi-do 13620 Republic of Korea; 3 Department of Surgery, Seoul National University Bundang Hospital, 173-82 Gumi-ro, Bundang-gu, Seongnam-si, Gyeonggi-do 463-707 Republic of Korea; 4 Department of Pathology, Seoul National University College of Medicine, 103 Daehak-ro, Jongno-gu, Seoul, 03080 Republic of Korea

## Abstract

We focused on the utility of the droplet digital polymerase chain reaction (ddPCR) for detecting *c-MYC* gene copy number (GCN) gain in cell-free plasma and tumor tissue of colorectal cancer (CRC) patients. *c-MYC* GCN status was determined using dual-color silver *in situ* hybridization (SISH) and ddPCR in retrospective cohort 1 (192 CRC patients) and prospective cohort 2 (64 CRC patients). In cohort 1, *c-MYC* GCN gain was observed in 34 (17.5%) patients by SISH, and in 7 (3.6%) patients by ddPCR. *c-MYC* GCN by SISH significantly correlated with ddPCR results (ρ = 0.532, P < 0.001). Although 40 cases (20.7%) showed intratumoral genetic heterogeneity, it did not cause discordance in results obtained by the two methods. *c-MYC* GCN gain, by both SISH and ddPCR was independently correlated with worst prognosis (P = 0.002). In cohort 2, *c-MYC* GCN estimation in tissue by ddPCR was also significantly associated with results obtained by SISH (ρ = 0.349, P = 0.005), but correlated with plasma ddPCR with borderline significance (ρ = 0.246, P = 0.050). Additionally, detecting *c-MYC* GCN gain in plasma with ddPCR might have relatively low sensitivity but high specificity. Our study suggests that ddPCR can be a useful tool for detecting *c-MYC* GCN gain as a potential prognostic biomarker in CRC tissue samples; however, this will need further verification in plasma samples.

## Introduction

The *c-MYC* gene encodes the c-MYC protein, which acts as a transcription factor for tumorigenesis in various cancers^1^. It has a critical role, especially in colorectal cancer (CRC) progression^2^ and has been identified as a target gene in *APC* signaling pathway in CRC^3^. Moreover, gene copy number (GCN) gain of *c-MYC* has been reported to be a common mechanism of resistance to various chemotherapies^4^. We previously reported the use of a silver *in situ* hybridization (SISH) method for detection of *c-MYC* GCN gain as a prognostic marker in CRC patients^5^. In the present study, we focused on the potential utility of droplet digital polymerase chain reaction (ddPCR) in detecting *c-MYC* GCN gain in tumor tissue and cell-free plasma of CRC patients, as a prognostic marker.

Circulating tumor DNA (ctDNA) has emerged as a potential tumor source for non-invasive diagnosis of cancer^6^. Therefore, liquid biopsy has gained importance in oncology, as a new approach that might overcome the need for invasive tissue biopsy^7,8^. Tissue biopsy comes with the limitations of technical and spatial heterogeneity, depending on the locus of metastatic or relapsed cancer. It might contain only a single lesion from a genetically heterogeneous tumor and hence miss newly acquired genetic aberrations in it^9,10^. On the other hand, liquid biopsy is considered capable of detecting genetic alterations that are partially acquired after treatment.

However, analysis of ctDNA requires a method of high sensitivity, as tumor DNA is present at a very low concentration in plasma; thus, ddPCR is expected to overcome this limitation^11^. This method has better ability to precisely quantify the concentration of DNA in a sample as compared to that of traditional quantitative PCR. The ddPCR has been reported to detect cancer mutational status with high concordance^12–14^. Interestingly, previous studies have indicated that ddPCR has the ability to accurately screen for GCN status as well as mutations in plasma DNA^15^. Analysis of ctDNA with ddPCR has the potential to detect *HER2* amplification in breast and stomach cancer^16,17^. Moreover, it has been shown that ddPCR can determine the *MET* GCN status in ctDNA with high accuracy^18^. Therefore, ddPCR seems to be a suitable and highly sensitive technique for GCN detection in ctDNA.

In this study, we aimed to analyze whether ddPCR could be adapted to detect small increases of *c-MYC* GCN in plasma and compared with the *c-MYC* GCN detected in the primary CRC tissue, using SISH and ddPCR.

## Results

### Clinicopathological features and frequency of *c-MYC* GCN gain in cohort 1

We investigated *c-MYC* GCN in 192 CRC tissues of cohort 1 by two different methods: SISH and ddPCR. *c-MYC* GCN gain, defined as mean *c-MYC* copies/nucleus ≥ 4.0 in SISH analysis, was observed in 34 (17.5%) cases, while by ddPCR method, was observed in 7 (3.6%) cases. Despite the discordance in frequency between the two methods, results by these two methods were significantly associated by Pearson’s correlation (ρ = 0.532, P < 0.001).

We hypothesized that the genetic heterogeneity of *c-MYC* GCN in each tumor cell might be the cause of discrepancy between the SISH and ddPCR results. Intratumoral genetic heterogeneity of *c-MYC* GCN gain, which was arbitrarily defined as the tumor cells with *c-MYC* GCN ≥ 4.0, consisted 5 to 50%. When the cells with *c-MYC* GCN ≥ 4.0 were less than 5% or more than 50%, the tumor was considered genetically homogenous in terms of *c-MYC* GCN. Forty cases (20.8%) showed intratumoral genetic heterogeneity for *c-MYC* GCN gain. However, intratumoral genetic heterogeneity of *c-MYC* GCN gain was not causal for the discordance in results between SISH and ddPCR methods (Table 1; P = 0.492).

**Table 1:** The correlation between concordance of SISH and ddPCR result, and intratumoral genetic heterogeneity in 192 CRC patients of cohort 1.

		Concordance of SISH and ddPCR result			
		Concordance	Discordance	Total	P value
Intratumoral genetic heterogeneity	Homogeneous	126 (65.6%)	26 (13.5%)	152 (79.2%)	0.492
	Heterogeneous	35 (18.2%)	5 (2.6%)	40 (20.8%)	
	Total	161 (83.9%)	31 (16.1%)	192 (100%)	

p value is from the χ^2^ or Fisher’s exact test and were significant at less than 0.05.

Table 2 summarizes the correlations detected between clinicopathological features and *c-MYC* GCN gain by ddPCR, in cohort 1. Since we have previously demonstrated the correlation between *c-MYC* GCN gain by SISH, and clinicopathological features of CRC^5^, here we present only the results of the ddPCR analysis. However, no statistically significant correlation was observed between the clinicopathological factors and *c-MYC* GCN gain by ddPCR.

**Table 2:** The correlation between clinicopathological factor and *c-MYC* GCN gain with ddPCR in 192 CRC patients of cohort 1.

		Total number of cases	*c-MYC* ddPCR				p-value
			4 > GCN		4 ≤ GCN		
Age	60>	114	110	59.5%	4	57.1%	0.903
	60≤	78	75	40.5%	3	42.9%	
Sex	Male	60	59	31.9%	1	14.3%	0.324
	Female	132	126	68.1%	6	85.7%	
Size	5 cm>	86	83	44.9%	3	42.9%	0.916
	5 cm≤	106	102	55.1%	4	57.1%	
Location	Ascending to descending colon	71	70	37.8%	1	14.3%	0.205
	Recto-sigmoid colon	121	115	62.2%	6	85.7%	
T stage	1–2	29	28	15.1%	1	14.3%	0.951
	3–4	163	157	84.9%	6	85.7%	
N stage	0	89	87	47.0%	2	28.6%	0.336
	1–2	103	98	53.0%	5	71.4%	
M stage	0	151	147	79.5%	4	57.1%	0.157
	1	41	38	20.5%	3	42.9%	
Differentiation	Low grade	169	163	88.1%	6	85.7%	0.848
	High grade	23	22	11.9%	1	14.3%	
Lymphatic invasion	No	79	78	42.2%	1	14.3%	0.141
	Yes	113	107	57.8%	6	85.7%	
Venous invasion	No	139	135	73.0%	4	57.1%	0.171
	Yes	53	50	27.0%	3	42.9%	
Perineura invasion	No	150	146	78.9%	4	57.1%	0.358
	Yes	42	39	21.1%	3	42.9%	
Tumor border	Infiltrative	21	21	11.4%	0	0.0%	0.345
	Expensile	171	164	88.6%	7	100.0%	
MSI status	MSS/MSI-L	163	154	86.0%	7	100.0%	0.288
	MSI-H	25	25	14.0%	0	0.0%	
Post-operative Chemotherapy	No	49	45	24.3%	4	57.1%	0.051
	Yes	143	140	75.7%	3	42.9%	

p values are from the χ^2^ or Fisher’s exact test and were significant at less than 0.05.

### Overall survival of cohort 1 patients with *c-MYC* GCN gain by SISH and ddPCR methods

**Figure 1:**
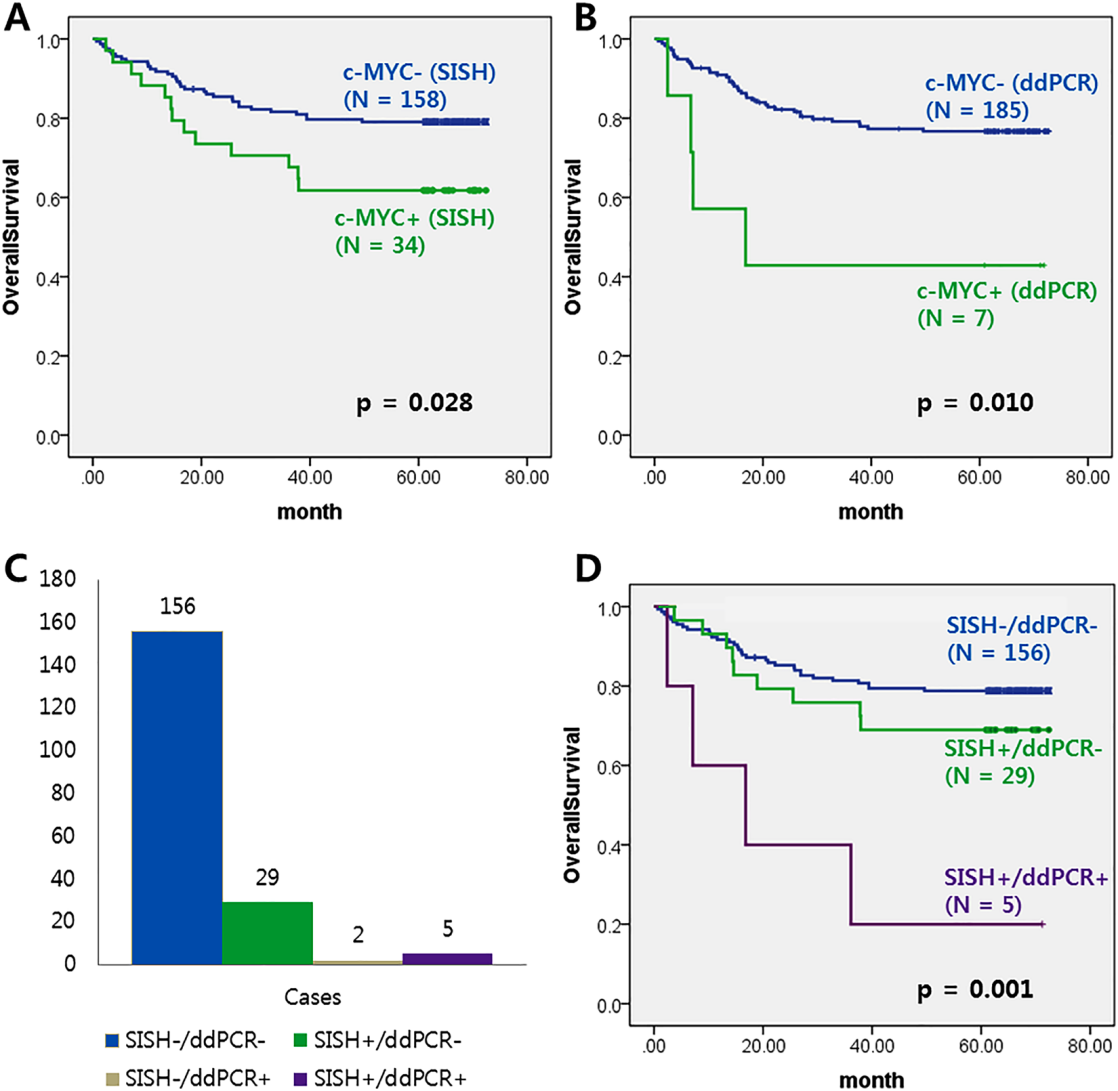
The *c-MYC* GCN gain status and overall survival in 192 CRC patients of cohort 1. Kaplan–Meier survival curves illustrating the prognostic effect of *c-MYC* GCN gain with detection methods. (**A**) *c-MYC* GCN gain by SISH was associated with unfavorable overall survival. (**B**) *c-MYC* GCN gain by ddPCR was associated with unfavorable overall survival. (**C**) No *c-MYC* GCN gain by either SISH or ddPCR, *c-MYC* GCN gain by only SISH, *c-MYC* GCN gain by only ddPCR, and *c-MYC* GCN gain by both SISH and ddPCR was found in 156 (81.3%), 29 (15.1%), 2 (1.0%) and 5 (2.6%) cases respectively. (**D**) *c-MYC* GCN gain by both SISH and ddPCR was correlated with unfavorable prognosis.

Kaplan–Meier survival curves illustrated the prognostic effect of *c-MYC* GCN gain by different detection methods. The mean overall survival of patients with CRC of cohort 1 was 55 months (range 1–73 months). Regardless of the detection method, *c-MYC* GCN gain was associated with unfavorable overall survival in primary CRC tissues (Fig. 1A,B; P = 0.028 and P = 0.010, respectively). In Fig. 1C, *c-MYC* GCN gain by neither SISH nor ddPCR (SISH-/ddPCR-), by only SISH (SISH+/ddPCR-), by only ddPCR (SISH-/ddPCR+), and by both SISH and ddPCR (SISH+/ddPCR+), was found in 156 (81.3%), 29 (15.1%), 2 (1.0%) and 5 (2.6%) cases respectively. We attempted to perform SISH on whole tissue sections from two SISH-/ddPCR+ cases to determine the reason for the discordant results. However, we did not observe *c-MYC* GCN gain (SISH ≥ 4) in whole sections of these tumors. *c-MYC* GCN gain by both SISH and ddPCR (SISH+/ddPCR+) was most significantly correlated with unfavorable prognosis (Fig. 1D; P = 0.001). As there were only two SISH-/ddPCR+ cases, this was not a sufficient number for performing survival analysis.

In addition, multivariate Cox proportional hazards analysis indicated that *c-MYC* GCN gain with both SISH and ddPCR (SISH+/ddPCR+) independently predicted unfavorable prognosis in cohort 1 (Table 3).

**Table 3:** Multivariate Cox proportional hazard models for predictors of overall survival in cohort 1 patients.

Factors	Univariate survival analysis		Multivariate survival analysis	
	HR (95% CI)	*P* value	HR (95% CI)	*P* value
***c-MYC*** **GCN gain in both SISH and ddPCR (SISH**+**/ddPCR**+**)**	**5.807** (**2.072–16.276)**	**0.001**	**6.067** (**1.912–19.258)**	**0.002**
Venous invasion	5.292 (2.957–9.470)	<0.001	1.476 (0.750–2.904)	NS (0.256)
Differentiation (low grade vs. high grade)	3.619 (1.869–7.008)	<0.001	2.503 (1.212–5.170)	0.013
T stage (1–2 vs. 3–4)	9.051 (1.247–65.675)	0.029	2.395 (0.300–19.139)	NS (0.410)
N stage (0 vs. 1, 2)	5.658 (2.529–12.659)	<0.001	1.770 (0.701–4.469)	NS (0.227)
M stage (0 vs. 1)	14.758 (7.789–27.964)	<0.001	8.882 (4.145–19.031)	<0.001

### Clinicopathological characteristics of cohort 2 patients

Table 4 demonstrates the relationships between *c-MYC* status (tissue ddPCR) and the clinicopathological parameters of the patients of cohort 2. For analyzing ctDNA, it is necessary to focus on clinicopathological factors that presumably account for high concentrations of ctDNA in plasma. At the time of diagnosis, although only two cases showed distant metastasis, 30 cases had lymph node involvement and 11 cases were T4 stage. Lymphatic and venous invasion was observed in 44 and 16 cases, respectively.

**Table 4:** The correlation between clinicopathological factor and *c-MYC* GCN gain with ddPCR in 64 CRC patients of cohort 2.

		Total number of cases	*c-MYC* ddPCR (Tissue)				p-value
			4 > GCN		4 ≤ GCN		
Age	60>	39	37	61.7%	2	50.0%	0.643
	60≤	25	23	38.3%	2	50.0%	
Sex	Male	23	22	36.7%	1	25.0%	0.638
	Female	41	38	63.3%	3	75.0%	
Size	5 cm>	49	47	78.3%	2	50.0%	0.195
	5 cm≤	15	13	21.7%	2	50.0%	
Location	Ascending to descending colon	17	14	23.3%	3	75.0%	0.023
	Recto-sigmoid colon	47	46	76.7%	1	25.0%	
T stage	1–2	17	16	26.7%	1	25.0%	0.942
	3–4	47	44	73.3%	3	75.0%	
N stage	0	34	34	56.7%	0	0.0%	0.028
	1–2	30	26	43.3%	4	100.0%	
M stage	0	62	58	96.7%	4	100.0%	0.711
	1	2	2	3.3%	0	0.0%	
Differentiation	Low grade	56	54	90.0%	2	50.0%	0.019
	High grade	8	6	10.0%	2	50.0%	
Lymphatic invasion	No	44	43	71.7%	1	25.0%	0.051
	Yes	20	17	28.3%	3	75.0%	
Venous invasion	No	48	46	76.7%	2	50.0%	0.233
	Yes	16	14	23.3%	2	50.0%	
Perineura invasion	No	39	37	61.7%	2	50.0%	0.643
	Yes	25	23	38.3%	2	50.0%	
Tumor border	Infiltrative	14	14	23.3%	0	0.0%	0.274
	Expensile	50	46	76.7%	4	100.0%	
Post-operative Chemotherapy	No	56	52	86.7%	4	100.0%	0.435
	Yes	8	8	13.3%	0	0.0%	

p values are from the χ^2^ or Fisher’s exact test and were significant at less than 0.05.

*c-MYC* GCN gain (tissue ddPCR) may be correlated with tumor a location of the ascending to descending colon (P = 0.023) and lymph node metastasis (P = 0.028). Histologically, low-grade CRCs seem to lack the *c-MYC* GCN gain (P = 0.019). However, only four cases showed *c-MYC* GCN gain (tissue ddPCR), which was not sufficient for a statistically significant result from the χ^2^ or Fisher’s exact test.

### Comparative analysis of *c-MYC* GCN status of tumor tissue and plasma ctDNA in cohort 2

**Figure 2:**
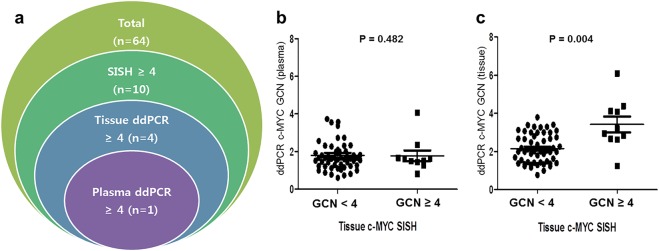
Comparative analysis of *c-MYC* GCN status between tumor sample and plasma cell-free DNA (cfDNA) in 64 CRC patients of cohort 2. (**a**) Frequency of *c-MYC* GCN gain by different detection methods. (**b**) Comparison between cfDNA (ddPCR) and tumor sample (SISH); (**c**). Comparison between tumor sample (ddPCR) and tumor sample (SISH).

In cohort 2, investigation of *c-MYC* GCN status was conducted on plasma sample as well as on surgical specimens of 64 CRC patients. *c-MYC* GCN gain by SISH, was observed in 10 (15.6%) patients, by tissue ddPCR in four (6.3%) patients and by plasma ddPCR in one (1.6%) patient. A patient who was detected with *c-MYC* GCN gain by plasma ddPCR, also showed *c-MYC* GCN gain by tissue ddPCR and SISH. All four *c-MYC* GCN gain cases by tissue ddPCR, also showed *c-MYC* GCN gain by SISH (Fig. 2a).

Intratumoral genetic and regional (central and peripheral) heterogeneity of *c-MYC* status was found in 29 (45.3%) and 8 (12.7%) cases, respectively. However, these heterogeneities were not causal for the discrepancy in *c-MYC* status results obtained, via different methods of detection (P > 0.05, Supplementary Table 1).

Table 5 demonstrates the correlation coefficient of *c-MYC* statuses obtained by different methods of determination. *c-MYC* GCN by SISH was significantly associated with tissue ddPCR results (ρ = 0.349, P = 0.005), but not with plasma ddPCR results (P = 0.620). *c-MYC* status in plasma was positively associated with tissue ddPCR results but showed only borderline statistical significance (ρ = 0.246, P = 0.050). Moreover, *c-MYC* GCN gain (≥4.0) by SISH was significantly associated with tissue ddPCR results (P = 0.004), but not with plasma ddPCR results (P = 0.482) in Wilcoxon rank-sum test (Fig. 2b,c).

**Table 5:** The correlation coefficient of detecting *c-MYC* GCN by different methods in 64 CRC patients of cohort 2.

	Pearson Correlations (P value)		
Cohort 2 (No = 64)		Tissue c-MYC ddPCR	Plasma c-MYC ddPCR
	c-MYC SISH	0.349 (P = 0.005)	0.037 (P = 0.620)
	Tissue c-MYC ddPCR	1	0.246 (P = 0.050)

## Discussion

The availability of noninvasive assays to detect and monitor tumor status is a major challenge in oncology. Although, ctDNA has emerged as a potential surrogate for precision medicine, the low levels of ctDNA pose a big challenge for successful detection. Recent studies have suggested that the discordance in detection rate between tumor tissue and plasma can be resolved by high-sensitivity ddPCR^19–22^. Representatively, the ddPCR assay for *EGFR* mutation in lung cancer was reported to be a highly sensitive and specific biomarker for clinical blood testing^23,24^. In CRC, the clinical utility of ddPCR to detect *KRAS* mutation in ctDNA is thought to be promising^14,25^.

Furthermore, approaches for detection of GCN alteration from ctDNA are also under the spotlight^15,26–28^. Bhuvan *et al*. suggested that point mutations in ctDNA might be difficult to detect due to the low ctDNA concentration derived from early stage cancer^27^. On the contrary, GCN gain can contribute a much larger number of ctDNA fragments to the overall plasma. Hence, detection of GCN gain might hypothetically be easier than that of point mutations in ctDNA-based cancer screening. Studies on *HER2* have been the most active in the field of detecting GCN gain in ctDNA, because *HER2* is clinically important in patients with breast cancer and gastric cancer. Kinugasa *et al*. reported that the concordance rate of ctDNA with formalin-fixed and paraffin-embedded (FFPE) tissue was not high (62.5%) in gastric cancer^29^. However, Katsutoshi *et al*. reported that the preoperative plasma *HER2* ratio correlated with the tumor *HER2* status, and sensitivity and specificity were 0.733 and 0.933, respectively, in gastric cancer patients^16^. Heidrun *et al*. indicated that ctDNA is a potential screening tool for *HER2* amplification in metastatic breast cancer with a positive and negative predictive value of 70% and 92%, respectively^17^. Although ddPCR for detecting *HER2* GCN status in ctDNA may be relatively less sensitive, its specificity seems to be high. In our study, we observed borderline correlation between plasma and tissue *c-MYC* GCN status by ddPCR. However, there was no significant correlation between *c-MYC* GCN status in plasma by ddPCR, and in tissue by SISH (Table 5). Interestingly, only one case was detected with *c-MYC* GCN gain (*c-MYC* ≥ 4) in plasma. This case also showed *c-MYC* GCN gain in tissue by both ddPCR and SISH (Fig. 2), indicating the reliability of the ddPCR assay to detect *c-MYC* GCN gain in ctDNA. Detecting *c-MYC* GCN gain in ctDNA might have low sensitivity and relatively high specificity. Moreover, all the cases that were detected with *c-MYC* GCN gain in tissue by ddPCR, also showed *c-MYC* GCN gain by SISH.

Despite reports of higher frequency of detectable ctDNA in CRC compared to other cancers^6^, detection of *c-MYC* GCN in plasma by ddPCR was found to be limited by low sensitivity in our study. The probable reason for this could be the difference in clinical characteristics of the participating patients of the various studies. Heidrun *et al*. reported mainly on metastatic breast cancer^17^, and the study by Katsutoshi *et al*. consisted of patients with high rates of lymph node metastasis (83%) and T4 stage (53%)^16^. Our study consisted of only 2 (3.6%) cases with distant metastasis, 30 (46.9%) cases with lymph node involvement, and 11 (17.2%) cases with T4 stage. Relatively early stage CRC patients were included and hence the quantity of released ctDNA in the plasma might have been insufficient for detection. Indeed, sensitivity might prove to be a limitation in detecting *c-MYC* GCN status in ctDNA of non-advanced CRC patients.

Focusing on FFPE tissue, previous research demonstrated that ddPCR method was as effective as fluorescence *in situ* hybridization (FISH) and therefore can become a standard method^29–31^. Our study demonstrated that results from SISH positively correlated with the results from ddPCR in FFPE tissues, of both cohort 1 and 2 (ρ = 0.532, P < 0.001 and ρ = 0.349, P = 0.005). Nonetheless, when the GCN gain criteria (*c-MYC* ≥ 4) are applied, the frequency of GCN gain is observed to be discordant, depending on the detection method used. Several reasons for this discordance can be suggested: first, FFPE tissues require fixation, and this may cause genomic DNA damage and degradation. These conditions can induce false negative results because of the low quality and quantity of DNA. Second, SISH is a microscopy-based method that involves directly and optically counting the target gene in tumor cells. This may be the most accurate method; however, personal observation and manual calculation can be potentially error-prone. We cannot exclude the possibility therefore that the SISH method produces more false positive results than ddPCR. On the other hand, the determination of GCN by ddPCR might be underestimated due to the presence of non-tumor cells, including immune cells and stromal cells in the sample^18,26^. A recent study recommended estimating the tumor content ratio (TCR) of a sample for improving the accuracy of GCN analysis by ddPCR^32^. They suggested that determining the *HER2* status using ddPCR, calibrated by the TCR, is advisable in clinical practice because non-tumor cells can influence the GCN status in samples with a relatively small amount of cancer cells. The tumor fraction of our samples might have influenced the *c-MYC* GCN detection by ddPCR. This may be the main reason that GCN gain (*c-MYC* ≥ 4) by ddPCR was less frequently observed than that by SISH.

Two cases showed *c-MYC* GCN gain only by ddPCR (SISH-/ddPCR+), whereas 29 cases showed *c-MYC* GCN gain only by SISH (SISH+/ddPCR-) in cohort 1 (Fig. 1C). We attempted to discover the reason for discordance between the two methods and hypothesized that this discordance could be induced by intratumoral genetic heterogeneity. If there was intratumoral heterogeneity of *c-MYC* GCN, an amplified portion would be missed in the SISH test or non-amplified portion would cause the ddPCR result to be negative for *c-MYC* GCN gain. The intratumoral heterogeneity of *c-MYC* GCN was arbitrarily defined as GCN gain (*c-MYC* ≥ 4) in tumor cells between 5% and 50%. Indeed, by SISH microscopy, we detected intratumoral genetic heterogeneity of *c-MYC* GCN in each tumor cell. However, we were unable to find significant association between discordant results depending on methods and intratumoral heterogeneity of *c-MYC* GCN.

We previously reported that *c-MYC* GCN gain with SISH is a poor prognostic marker for CRC patients^5^. In the present study, *c-MYC* GCN gain was correlated with unfavorable overall survival, not only by SISH but also by ddPCR. Interestingly, *c-MYC* GCN gain with both SISH and ddPCR (SISH+/ddPCR+) was independently correlated with worst prognosis (Table 3).

In conclusion, we tried to determine the *c-MYC* GCN status in the ctDNA of preoperative CRC patients, by ddPCR. To the best of our knowledge, we are the first to attempt detecting *c-MYC* GCN by ddPCR in CRC patients. Although the ddPCR assay showed low sensitivity in detecting *c-MYC* GCN gain in ctDNA of non-advanced CRC patients, it detected *c-MYC* GCN gain in ctDNA with high specificity. However, we cannot recommend ddPCR of plasma samples as a first screening tool for *c-MYC* GCN gain due to the high risk of false negative results. Thus, ddPCR may require further evaluation in plasma samples. There was also discrepancy between *c-MYC* GCN gain measured by SISH and ddPCR in FFPE tissues; nevertheless, the ddPCR results were significantly correlated with the SISH results. Thus, we can suggest the detection of *c-MYC* GCN gain by ddPCR as a potential prognostic biomarker in CRC tissue.

## Materials and Methods

### Patients and samples

We collected tissue samples from 192 CRC patients who underwent surgery between Jan 2006 to Dec 2006, at the Seoul National University Bundang Hospital (cohort 1). Additionally, to evaluate the *c-MYC* GCN gain in plasma, we prospectively recruited a cohort of 64 CRC patients, who had undergone surgery between Mar 2011 and Mar 2012 (cohort 2). Plasma samples were collected from all cohort 2 patients, 1–20 days before operation. FFPE tumor tissue was also collected from all cohort 2 patients. Patients who had received pre-operative chemotherapy or radiotherapy were excluded from the cohort. Pathologists K.S.L and H.S.L reviewed all the cases. Cancer stage was determined from the American Joint Committee on Cancer (AJCC), 7th edition. Clinical and pathological information was acquired from the hospital medical records, including patient outcome and survival. The Institutional Review Board of Seoul National University Bundang Hospital (reference: B-1012/117-011) approved the use of medical record data, patient tissue and plasma samples for this study. Informed consent was obtained from all participants of cohort 2 and exempt from being required from in participants of cohort 1. Our ethics committee has waived informed consent for retrospective research using tissue samples obtained before 2012. All methods were performed in accordance with the relevant guidelines and regulations.

### Tissue array method

Surgically resected primary CRC tumor samples were fixed in formalin and embedded in paraffin. Two-millimeter core tumor tissue samples were obtained from each donor block and rearranged in a new recipient tissue microarray block. In addition, to evaluate the regional heterogeneity of *c-MYC* GCN gain, we obtained tumor samples from each central and peripheral lesion in 64 CRC patients of cohort 2. Each core containing >30% tumor cells were considered valid cores.

### Dual-color silver *in situ* hybridization

The *c-MYC* gene was visualized by using a blue-staining system (ultraView silver *in situ* hybridization [SISH] dinitrophenol [DNP] detection kit and c-MYC DNP probe, Ventana Medical Systems, Tucson, AZ, USA). The centromere of chromosome 8 (CEP8) was visualized by using a red-staining system (ultraView red ISH digoxigenin [DIG] detection kit and chromosome 8 DIG probe, Ventana Medical Systems). Positive signals were visualized at 60× magnification and counted in 50 non-overlapping tumor cell nuclei for each case. Small and large clusters were scored as 6 and 12 signals, respectively. A *c-MYC* GCN gain ≥ 4 copies/cell was observed to be the most predictive cut-off point for patient prognosis in a previous study^5^.

### DNA isolation from tumor samples

DNA was extracted from FFPE tumor specimens. The represent area contained >30% tumor cells. The corresponding areas were marked on 4 slide sections (8 μm). Tissue sections were deparaffinized by the boiling method with incubation at 70 °C for 10 minutes and centrifugation for 10 minutes at maximum speed^33^. After deparaffinization, DNA extraction was performed using QIAamp DNA FFPE Tissue Kit (Qiagen, Hilden, Germany) according to the manufacturer’s instructions. The DNA digestion procedure was skipped in this study because the DNA from FFPE tissues was already fragmented.

### Preparation of plasma and extraction of circulating DNA

Blood samples were processed within 2 hours of collection and centrifuged at 3000 rpm for 10 min. Plasma was filtered using Fisherbrand standard Serum Filter (13 mm × 4″) (Fisher HealthCare, Huston, TX, USA) before DNA extraction. DNA was extracted from 300 μL of plasma, using the High Pure viral Nucleic Acid Kit (Roche, Branchburg, NJ, USA) according to the manufacturer’s instructions. The DNA digestion procedure was also skipped for plasma samples because of the small amount of plasma DNA. The manufacturer’s protocol recommends digestion when the DNA input is greater than 75 ng.

### Droplet digital PCR

Using the human eukaryotic initiation factor 2C1 (*EIF2C1*) gene as an internal control to assess the copy number of the *MYC* gene, *MYC*-to-*EIF2C1* ratios were determined using ddPCR. Each sample was partitioned into 20,000 droplets, with target and background (reference) DNA distributed randomly, but not uniformly, among the droplets. Amplicon lengths for ddPCR reaction of *MYC* and *EIF2C1* were 121 bp and 86 bp, respectively. The following FAM probes were used for ddPCR; *MYC*: PrimePCR™ ddPCR™ Copy Number Assay (Bio-Rad) and HEX probes for *EIF2C1*: PrimePCR™ ddPCR™ Copy Number Assay (Bio-Rad). The reactions were performed in 20 μL reaction volumes that consisted of up to 50 ng of extracted DNA (1 μL), 2x ddPCR supermix for probes (No dUTP) (10 μL), *MYC* primers/probes (1 μL), *EIF2C1* primer/probes (1 μL) and deionized distilled water (7 μL). Emulsified PCRs were run in a 96-well plate on a C1000 Touch™ Thermal Cycler (Bio-Rad). The plates were incubated at 95 °C for 10 min, followed by 50 cycles of 94 °C for 30 s, 60 °C for 60 s and 10 min incubation at 98 °C. The plates were read on a Bio-Rad QX200 droplet reader using the QuantaSoft v1.7.4 software (Bio-Rad) to assess the number of droplets positive for *MYC* and/or *EIF2C1*. *MYC* gene copy number determined by ddPCR was defined as 2 × *MYC*/*EIF2C1*. The cut-off for classifying samples as *MYC* GCN gain was set as ≥ 4 copies/cell. In addition, positive and negative experimental results were obtained according to the Digital MIQE Guideline and are shown in Supplementary Fig. 1.

### Microsatellite instability

Microsatellite instability (MSI) was determined by fragment analysis using an automated DNA sequencer (ABI 3731 Genetic Analyzer; Applied Biosystems, Foster City, CA, USA) with the following five microsatellite markers, according to previously described methods: BAT-26, BAT-25, D5S346, D17S250, and D2S123^34^.

### Statistical analyses

Categorical variables were compared using the Chi-square or Fisher’s exact test, as appropriate. The correlation between *c-MYC* GCN statuses via different methods was analyzed by Pearson’s correlation coefficients. The Wilcoxon rank-sum test was used to compare between *c-MYC* GCN gain results obtained by ddPCR and SISH. The prognostic effect of *c-MYC* GCN gain by the different detection methods was evaluated using Kaplan-Meier curves with the log-rank test. A threshold of P < 0.05 was considered statistically significant. IBM SPSS statistics version 21 (IBM, Armonk, NY, USA) was utilized for all statistical analyses.

## Supplementary information

Dataset 1

## Data Availability

The datasets generated during and/or analyzed during the current study are available from the corresponding author on reasonable request.
