# Supplementary material for: Digital polymerase chain reaction for detecting c-MYC copy number gain in tissue and cell-free plasma samples of colorectal cancer patients
Source: Sci Rep. 2019 Feb 7;9:1611. doi: 10.1038/s41598-018-38415-4 (PMC6367402; doi:10.1038/s41598-018-38415-4)
Supplement: Supplementary file 1 — Dataset 1 [file 41598_2018_38415_MOESM1_ESM.pdf]

Supplementary Information

Digital polymerase chain reaction for detecting *c-MYC* copy number gain in tissue and cell-free plasma samples of colorectal cancer patients

Kyu Sang Lee[1], Soo Kyung Nam[1], Soo Hyun Seo[2], Kyoung Un Park[2], Heung-Kwon Oh[3], Duck-Woo Kim[3], Sung-Bum Kang[3], Woo Ho Kim[4], Hye Seung Lee[1,4*]

[1]Department of Pathology, Seoul National University Bundang Hospital, 173-82 Gumi-ro, Bundang-gu, Seongnam-si, Gyeonggi-do 13620, Republic of Korea;

[2]Department of Laboratory Medicine, Seoul National University Bundang Hospital, 173-82 Gumi-ro, Bundang-gu, Seongnam-si, Gyeonggi-do 13620, Republic of Korea

[3]Department of Surgery, Seoul National University Bundang Hospital, 173-82 Gumi-ro, Bundang-gu, Seongnam-si, Gyeonggi-do 463-707, Republic of Korea

[4]Department of Pathology, Seoul National University College of Medicine, 103 Daehak-ro, Jongno-gu, Seoul 03080, Republic of Korea

* *Corresponding author*: Hye Seung Lee

E-mail address: hye2@snu.ac.kr

Supplementary Table 1. The correlation between concordance of SISH and ddPCR result, and intratumoral genetic and regional heterogeneity in 64 CRC patients of cohort 2

		**Concordance of SISH and tissue ddPCR result**				**Concordance of SISH and tissue plasma ddPCR result**			
		Concordance	Discordance	Total	P value	Concordance	Discordance	Total	P value
**Intratumoral genetic heterogeneity**	Homogeneous	30 (46.9%)	5 (7.8%)	35 (54.7%)	0.139	28 (43.8%)	7 (10.9%)	35 (54.7%)	0.133
	Heterogeneous	28 (43.8%)	1 (1.6%)	29 (45.3%)		27 (42.2%)	2 (3.1%)	29 (45.3%)	
**Regional (central and peripheral) heterogeneity**	Homogeneous	50 (78.1%)	6 (9.4%)	56 (87.5%)	0.331	48 (75.0%)	8 (12.5%)	56 (87.5%)	0.892
	Heterogeneous	8 (12.5%)	0 (0%)	8 (12.5%)		7 (10.9%)	1 (1.6%)	8 (12.5%)	

p values are from the $\chi 2$ or Fisher's exact test and were significant at less than 0.05.

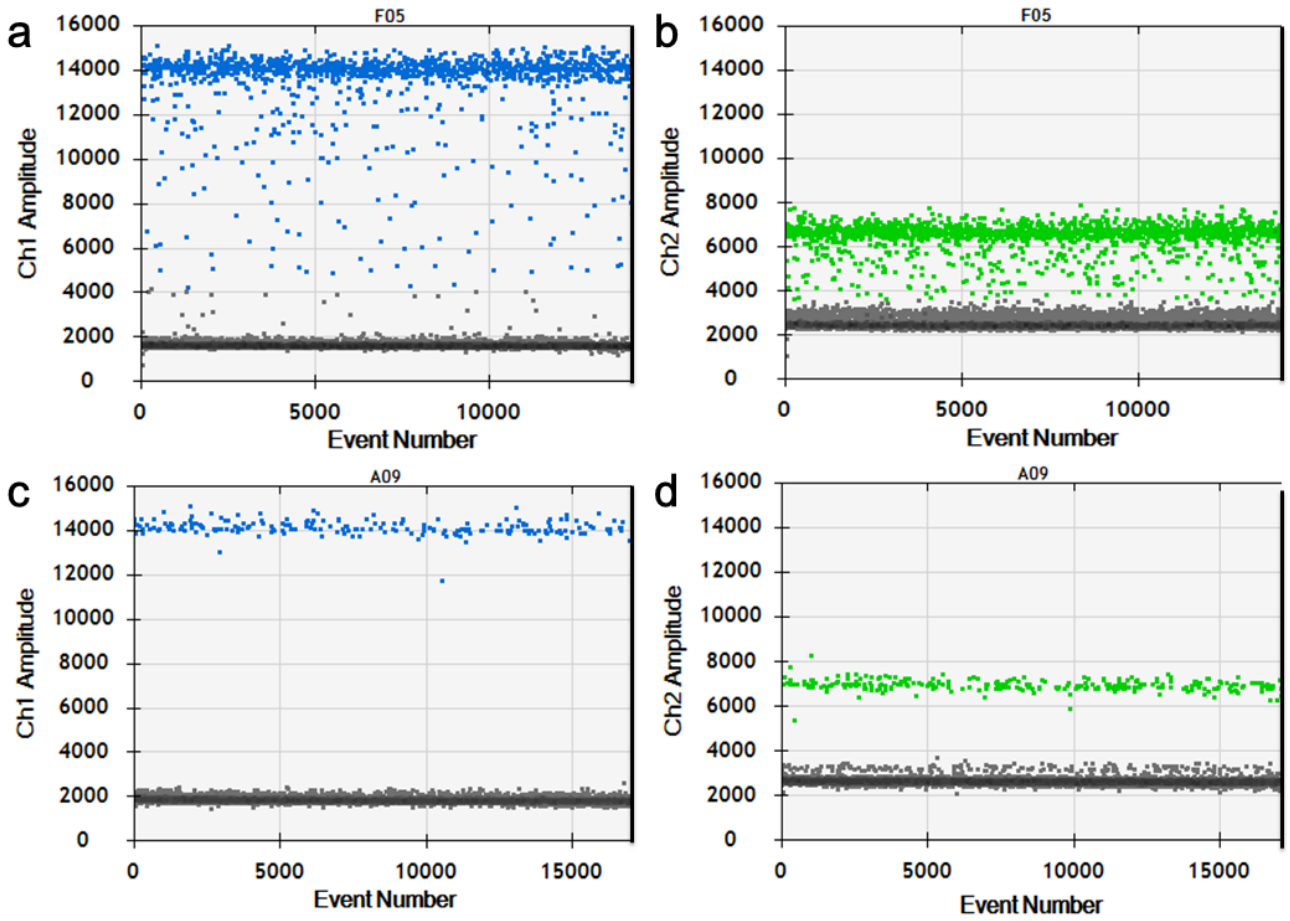

Supplementary Figure 1. Experimental positive and negative ddPCR results according to Digital MIQE Guideline. (a) Tissue distinction between positive and negative partitions FAM(MYC). (b) Tissue distinction between positive and negative partitions HEX(EIF2C1). (c) Plasma distinction between positive and negative partitions FAM(MYC). (d) Plasma distinction between positive and negative partitions HEX(EIF2C1).
